# Palliative and supportive care in patients with hepatocellular carcinoma: a qualitative study on attitudes and perceptions of health professionals

**DOI:** 10.1007/s00520-025-09403-y

**Published:** 2025-04-16

**Authors:** Cameron Gofton, Anna Di Bartolomeo, Shalini Wijekulasuriya, Shenghan Cai, Rose Boutros, Fiona Stafford-Bell, Kim Caldwell, Geoffrey McCaughan, Amany Zekry, Simone I Strasser, Miriam Levy, Caitlin Sheehan, Stephen Goodall, Jan Maree Davis, Linda Sheahan, Ken Liu, Sally Greenaway, Scott Davison, Thang Du Huynh, Zujaj Quadri, Yvonne A Zurynski, Meera Agar, Jacob George

**Affiliations:** 1 Storr Liver Centre, Westmead Hospital, WSLHD, Westmead Institute for Medical Research and University of Sydney, Sydney, Australia; 2 Royal North Shore Hospital, St Leonards, Australia; 3 Hepatology Services, NSLHD, St Leonards, Australia; 4 Centre for Healthcare Resilience and Implementation Science, Australian Institute of Health Innovation, Faculty of Medicine, Health and Human Sciences, Macquarie University, Sydney, Australia; 5 Department of Palliative Care, Liverpool Hospital, SWSLHD, Liverpool, Australia; 6 Department of Palliative Care, St George Hospital, SESLHD, Sydney, Australia; 7 A W Morrow Gastroenterology and Liver Centre, Royal Prince Alfred Hospital,, SLHD and University of Sydney, Camperdown, Australia; 8 Department of Gastroenterology and Hepatology, St George Hospital, SESLHD, Sydney, Australia; 9 Department of Gastroenterology and Hepatology, Liverpool Hospital, SWSLHD, Liverpool, Australia; 10 University of Technology Sydney, Ultimo, Australia; 11 Palliative Care Service, SESLHD Southern Sector, Kogarah, Australia; 12 Department of Palliative Care, Westmead Hospital, Westmead, Australia; 13 Australian Institute of Health Innovation, Macquarie University, Sydney, Australia

**Keywords:** Palliative care, Palliative and supportive care, Liver cancer, Hepatocellular carcinoma, Health professionals, Qualitative

## Abstract

**Background:**

Integration of palliative and supportive care in cancer treatment pathways is becoming standardised. While there has been significant qualitative research in oncology on palliative and supportive care integration into clinical care, there is little evidence that focusses on clinicians who manage hepatocellular carcinoma (HCC) and their perceptions on palliative and supportive care.

**Aim:**

To investigate the attitudes and perceptions regarding palliative and supportive care of healthcare professionals managing patients with HCC.

**Design:**

Qualitative study involving semi-structured individual interviews transcribed verbatim and analysed thematically.

Setting/participants.

A total of 25 healthcare professionals including hepatologists, gastroenterology trainees, hepatology clinical nurse consultants, social workers, and palliative care specialists providing care to patients with HCC recruited at 4 tertiary hospitals via purposive sampling.

**Results:**

The following themes emerged: (1) availability of palliative care services, (2) need for clear referral pathways and processes, (3) patients’ limited understanding of palliative care, (4) recognition of benefits of palliative care, and (5) the lack of training in hepatology services for palliative care provision.

**Conclusion:**

Health professionals’ perceptions of integration of palliative and supportive care in liver cancer care are hampered by multiple barriers. Opportunities to establish a more cohesive approach to care integration for patients with liver cancer have been identified.

**Trial Registration:**

ACTRN12623000010695 (date of registration 9/01/2023).

**Table:** 

What is already known about the topic?	
• Integration of palliative and supportive care for cancer can have profound benefits for patients’ symptom burden and quality of life.	
• There is a lack of empirical studies examining the perspectives of health professionals who manage liver cancer on the integration of palliative and supportive care into the treatment pathways for patients.	
What this paper adds	
• This study identifies a number of barriers to the implementation of palliative and supportive care into liver cancer treatment algorithms.	
• The absence of sufficient evidence in clinical guidelines impairs the capacity of health professionals to improve the integration of palliative and supportive care for liver cancer patients.	
Implications for practice, theory, or policy.	
• As liver cancer prevalence increases, further effort is required to develop appropriate evidence-based clinical guidelines and referral pathways to support the integration of palliative and supportive care within existing liver cancer services.

## Introduction

Hepatocellular carcinoma (HCC) is the sixth most common cancer and the second greatest contributor to cancer-related mortality [1]. The prognosis of HCC is poor with a 5-year survival of just 19%. Additionally, more than 75% of HCC patients have concurrent cirrhosis which also impacts on their long- term outcomes. HCC disproportionately affects marginalised populations including culturally and linguistically diverse (CALD) groups, refugees, and Indigenous populations [2–7]. Higher rates are also associated with substance abuse, high-risk sexual practices, and incarcerated populations [2–7]. HCC patients tend to have a fluctuating course between disease stabilisation, recurrence, and progression with multiple radiological, surgical, and systemic treatment modalities being utilised in a non-linear fashion. This non-linear natural history of HCC is often complex and varied in the context of underlying liver comorbidities, and this complexity hampers the ability of health professionals to formulate long-term individualised treatment plans.

International oncology guidelines have recently recommended the early integration of palliative and supportive care into treatment pathways, based on a number of seminal studies highlighting the importance of this practice [8–14]. Early integration of palliative and supportive services in the natural history of cancer has resulted in improvements in symptoms, quality of life, and in a few studies, a survival benefit [15–21]. Whilst these recommendations have been implemented in a number of different advanced cancers, clinical services for HCC are yet to integrate palliative and supportive care across the spectrum of the disease.

Although there is some evidence applying qualitative research methodology into oncologists’ perspectives on implementation of palliative and supportive care in malignancy, there is very little evidence in hepatology practice for clinicians who provide the primary management for patients with HCC [22]. The management of HCC varies across the world, with care falling either on oncologists or hepatologists. While some existing studies report the perceptions of hepatologists and other health professionals regarding their experiences with palliative and supportive care in managing liver disease, there are very few studies focusing on the attitudes, perceptions, and experiences of clinicians caring for patients with HCC [22].

The aim of this study was to investigate the attitudes, perceptions, and experiences of clinicians about involving palliative and supportive care services in the care of patients with HCC. This qualitative study examined the [1] health professionals’ views on patient perceptions and attitudes towards palliative and supportive care, [2] health professionals’ attitudes towards referrals of HCC patients to palliative and supportive care, and [3] perceived barriers or enablers to referral to palliative and supportive care.

## Methods

### Study design

A qualitative study using semi-structured interviews to investigate the attitudes, perceptions, and experiences of healthcare professionals about involving palliative and supportive care in the management of patients with HCC. The methods and reporting were informed by the consolidated criteria for reporting qualitative research (COREQ) guidelines [23].

### Participants and sampling

Participants were purposively sampled to ensure a diverse representation of professions and levels of seniority, whilst ensuring the recruitment of multiple professionals from each site. Ambulatory liver clinic leads and other clinical staff at each hepatology service including hepatologists, gastroenterology trainees, hepatology clinical nurse consultants, social workers, and palliative care physicians were invited to participate in individual interviews. Recruitment took place between September 2022 and November 2023.

### Setting

Four tertiary referral centres (ambulatory liver cancer clinics) in metropolitan Sydney, New South Wales (NSW), Australia, participated in the study. Each site is highly specialised in the management of patients with HCC and sees between 80 and 120 patients per year. The HCC services comprise diverse teams of multidisciplinary staff that service the needs of CALD populations across various geographic regions, encompassing a number of marginalised groups including Indigenous peoples, refugees, and people living with psychosocial adversity. One of the sites had embedded a palliative care clinician into the ambulatory HCC clinic. Ethical approval was obtained from the South Western Sydney Health District Human Research Ethics Committee on 25/05/2022 (ref no. 2022/ETH00385) in accordance with the Declaration of Helsinki.

### Data collection

Informed by previous peer-reviewed studies, semi-structured interviews were guided using an interview guide developed by the research team, based on preliminary discussions with palliative medicine consultants, hepatologists, and nurses.

Participants’ informed consents were collected and recorded at the beginning of each interview. One interviewer (C.G.) conducted all interviews. All interviews took place in a virtual setting, using Zoom for both video and audio recording. No repeat interviews were necessary. Participants were not remunerated.

### Data management and analysis

All interviews were transcribed verbatim by the interviewer (C.G.) and checked against audio–video recordings to ensure accuracy. Transcripts were then anonymised and deidentified. A numerical system was used to distinguish individuals from various study sites by codes for the site and participant number, e.g. P1.1 refers to site one, participant one. Thematic analysis was performed according to the methods described by Braun and Clarke [24]. This involved familiarisation with transcribed data, generating codes and themes using an inductive analysis approach, identifying emerging themes, and consolidating these into broad themes through team discussion, checking, and cross-checking against the original transcripts to remain true to the data. All transcripts were coded using NVivo 14 Pro software by three experienced qualitative researchers (S.W., S.C., Y.Z.) independently, to improve consistency of interpretation and minimise bias. Any discrepancies or uncertainties in coding and interpreting the data were discussed and resolved through mutual agreement. The associated codes were subsequently categorised into overarching themes that align with the research questions. Data extracts of transcripts under each theme were reviewed to ensure coherence and relevance and to identify any missed codes. Themes were then reviewed and discussed with the interviewer (C.G.) ensuring that the theme interpretation was informed by contextual information gleaned during the interview. Final themes were defined, and subthemes were identified. To capture potential variations in views among the different types of health professionals and professionals from different sites, the themes were analysed categorically based on both profession and site. As the results mostly did not differ by profession or by site, aggregated themes based on the whole group of respondents are reported. Any results that differed from the broad themes expressed by one or two individuals are highlighted.

### Reflexivity statement for researchers involved directly in qualitative data analysis

The lead researcher, CG, is an experienced gastroenterologist/hepatologist involved in the diagnosis and treatment of people with HCC including referral to palliative care, and their personal experience could potentially create a personal bias during analysis. Therefore, three other researchers (SW, SC, and YZ) undertook the thematic analysis. YZ is a senior academic and health services researcher with over 30 years of experience in health research and approximately 15 years of experience in qualitative research methods. SC is a demographer and a health services researcher with 4 years of post-doctoral experience and over 10 years of experience in mixed methods research. SW is an experienced research assistant with 2 years of experience of undertaking qualitative analyses related to health services research. SC, SW, and YZ were not involved in data collection and are not involved in clinical care; however, all three have previously conducted qualitative research involving interviews and focus groups with healthcare professionals, and all three also have personal experience of accessing the Australian healthcare system for their own healthcare or the care of relatives.

## Results

### Participant characteristics

Twenty-five of the 29 health professionals approached consented to participate in the interview (86%). Four health professionals either declined or were unable to participate. The duration of each Interview varied from 13 to 32 min with a median of 25 min. Participant characteristics are outlined in Table 1.

**Table 1:** Characteristics of interview participants

Characteristic	Healthcare Professionals (n=25)
Sex - Male - Female	9 (36%) 16 (74%)
Age - Median - Range	42 27-63
Profession - Hepatologist - Palliative Care Physician - Gastroenterology Trainee - Liver Cancer Clinical Nurse Consultant - Social Worker	14 (56%) 1 (4%) 4 (16%) 5 (20%) 1 (4%)

Overall, four broad themes were consistent across the four sites and among different types of health professionals:

1. Health professionals recognise the value of palliative care for their patients
2. Limited availability of and access to palliative care services
3. Need for clear referral pathways, processes, and for training
4. Clinician perceptions about patient attitudes to palliative care

### Theme 1: liver health professionals recognise the value of palliative care for patients

All participants perceived value in involving palliative care services in their patients’ care; however, they also talked about the need for closer interdisciplinary relationships (Table 2).

**Table 2:** Theme 1: benefits of palliative care identified by liver health professionals

Sub themes	Illustrative quote(s)
Currently the medical team does most management *Better interdisciplinary care needed* *Hepatology staff focused on medical care* *Interdisciplinary care provided* *Need outreach*	“We do see them very frequently and I feel like the medical team ends up managing a lot of the symptoms” (P1.1) “So actively supporting patients to engage with dietitians, engage them with a psychologist as well. This would be another way to ensure that patients, you know, have some independence for improving the health outcomes” (P1.8) “I think we don't tend to think about palliative care until it’s almost too late” (P1.5) “So, this sort of multi-disciplinary discussion that we have once a month for all complex cirrhotics, whether they need a transplant, or that we need psychology, social support. Basically, we enhance that. We like to havethe finger on the pulse. What’s happening with every patient?” (P3.4) “I have easier access to support in the community. Like occupational therapy, physiotherapy without having to admit the patients or linked to community Palliative care. As often these patients need a bit of community palliative care, they need sort of someone to do a home assessment” (P4.2)
Work closely with families	“Anyway, getting them engaged, their family members engaged knowing how to manage symptoms at home, improving the knowledge of the general practitioner as well, because that’s the first person to go to” (P4.5)
Work more closely with palliative care team	“It would be good to have some more clearer guidelines of when it can be introduced,and I think we should really have a better relationship with the palliative care team. They’ve just started coming to our MDT meetings, which is good. I think we need a better relationship with them and a clear referral pathway” (P4.2)

All healthcare professionals recognised the importance of palliative and supportive care and the potential benefits from the involvement of palliative care staff as early as feasible (Table 2). All participants acknowledged that the work of supporting HCC patients was multidisciplinary in nature; however, there was a strong sub-theme that most of the management responsibility falls within the scope of hepatologists’ core business with a focus on the medical treatment of HCC. Due to this medical management focus, there was a feeling amongst some participants that they were referring to palliative and supportive care too late in the patients’ journey, although the timing of referral was in keeping with international hepatology guidelines.

Most participants expressed a desire to have a closer working relationship with palliative and supportive care services to better manage liver cancer patients, especially when deciding at which point to introduce the involvement of palliative care specialists. The need to better integrate palliative and supportive care within the core multidisciplinary clinics caring for liver cancer patients was expressed by some healthcare workers in this study. Participants also felt that the involvement of palliative care teams facilitated closer relationships with patients and families as well as primary care clinicians who could support families in the community. The participants acknowledged the benefits that palliative care services had for patients with HCC and their family members.

### Theme 2: limited availability and access to palliative care services

Healthcare professionals described limited availability and capacity of palliative care services for their patients. They also recognised other access barriers such as geographical referral boundaries and limited knowledge about which palliative care services are available across individual local health networks (Table 3).

**Table 3:** Theme 2: limited availability and access to palliative care

Subthemes	Illustrative quote(s)
Availability of palliative care services	“Another factor that would influence might be also like the availability of palliative care and the types of services and programs that are available at that hospital network” (P1.1)
Access difficulties	“I guess up until this stage access to palliative care services was always quite difficult to get services because of the waiting list and lack of palliative clinical nurse consultants around so unless they’readmitted tohospital,we can’t actually get them seen or linkedwith, so I guess that's the main two barriers” (P3.3)
Geographical service boundaries	“Sometimes if, the address of the patient is beyond the catchment area of the local health district, then we have actually to start arranging the referral again to a different clinic and that might delay the process” (P2.4)
Limited capacity of palliative care staff	“In my experience sometimes, the capacity is quite stretched but they usually always try very hard to actually accommodate and try to see the patients” (P2.4)
No issues with palliative care relationship	“Well, we find that positive [palliative] care provides good support for our patients, so we don’t have major obstacles getting patients to be seen when we initiate a referral” (P2.5)
Referrals not made	“But in saying that I must be honest we don’t do that many palliative care referrals for these patients. We do see them very, very frequently and I feel like the medical team ends up managing a lot of the symptoms, where I'm sure actual palliative care input would be super helpful as well” (P1.1)
Variability across networks	“I think one of the difficulties is actually knowing if each service actually has the capacity to do and is willing to do. Because I think one of the complications,we have is that some services are better at managing the complications of the underlying liver disease. And others are extremely low. Because they don’t necessarily have the knowledge base to do that” (P4.6)

All participants acknowledged there was a perception of limited capacity within palliative care services within their networks. Most individuals stated that they encountered a variety of difficulties when trying to access palliative care services for their patients with HCC, often due to the limited availability of such services. Even when palliative care services are available, their ability to accommodate additional patient referrals is constrained by limited capacity and staff resources. Furthermore, outpatient palliative care services face constraints in capacity and staffing, resulting in limited availability for new patients, whereas the referral process is more streamlined for admitted patients.

There was a reported lack of consistency regarding referral pathways and processes, and many outlined that these problems arise due to varying systems, processes, and eligibility for referral being implemented at various levels throughout the health districts, hospitals, and across the state. Palliative care services may also have different geographical referral boundaries which creates confusion, delays, and additional work to find an alternative service. Participants highlighted that the services provided by palliative care providers varied in scope. The underlying drivers of this heterogeneous nature of service scope were not clear to the participants, but some suspected that it was due to resourcing restrictions and end point efficiency for palliative care. Due to the above reasons, some healthcare professionals indicated that they had deferred referrals of patients with HCC to palliative care, despite feeling that their patients would benefit from earlier referral.

Two hepatologists at different clinical sites had encountered no issues with referrals to palliative care and collaboration with staff from palliative care services once patients had been referred. It was unclear from the interviews what factors had influenced these participants’ perceptions.

### Theme 3: need for clear referral pathways, processes, and training

**Fig. 1:**
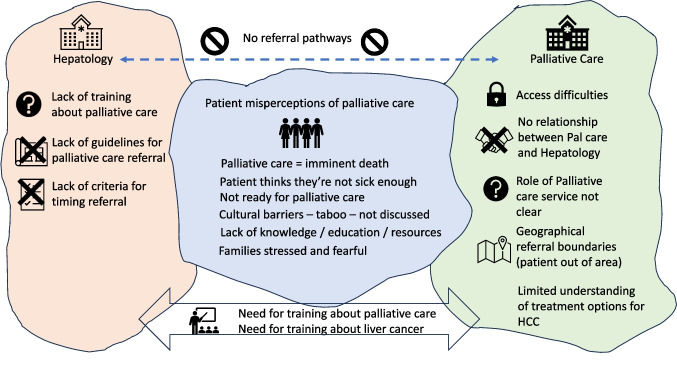
Interaction between hepatology, palliative care, and patients with HCC

All respondents talked about the need for more specific training of gastroenterologists/hepatologists in palliative care, and several mentioned the need for palliative care clinicians to have access to training in the natural progression, variability, and treatment of HCC (Table 4). They also identified referral process barriers and lack of clear referral guidelines (Fig. 1).

**Table 4:** Theme 3: identified needs for referral pathways, processes, and training

Subthemes	Illustrative quote(s)
Need for clear referral pathway and process	“The referral process needs to be streamlined and needs to be quite easy” (P2.4)
Existing clear referral pathways	“At the moment we have a consultant which is included which is linked to our liver service, which means she does clinic once a week with us as well, and that makes a referral to palliative care services much easier for a lot of patients. Most patients who have new diagnoses of HCCs are automatically linked to palliative care, and they get like an initial consultation with the palliative care consultant just to kind of like introduce themselves” (P3.3)
Lack of clear referral path to palliative care	“And I don’t really have a clear understanding of the pathways to involving palliative care at the moment or the work up as much as I should be” (P4.1)
Limited understanding among palliative care about treatment options	“If they’re receiving immunotherapy, they are having active treatment therefore they don’t qualify for palliative care. So, this is an area that I’ve had at least three refusals because they consider that is active treatment” (P1.2)
Need for guidelines *Criteria for referral not clear* • Also benefit if symptoms not severe • Need for referral criteria • Need for symptom monitoring Significant symptoms and limited HCC treatment options	“So rather than location, or even if it has something as a one-page document on the front page of the internet to tell us, this is the pathway and include practical information so you’re not wasting time sending referrals and ringing up to find out if they fulfill the criteria” (P1.2) “And I think there’s a whole grey zone in the middle. And within that grey zone is where it would be helpful to have some more guidance as to who would be best to be referred” (P1.1) “Maybe I can, I referred to, I don’t know. I don’t know why I’m doing this, but I’d like to think, maybe I'm referring them three to four weeks before [blind] Freddie can see that they need. Palliative Care, but maybe I’m wrong” (P1.3) “But I think it’s still relying heavily on us or the nurses telling them or updating them about the symptoms, because I guess we see them more often” (P3.3) **“**I think most of the patients that get referred the way in, it’s definitely symptoms for me. The main symptom, I would manage is usually pain” (P3.1)
Guidance for the timing of referral to palliative care	“And again, early referral when they don’t have symptoms is quite challenging if we’ve got them on therapy and they’re sort of under control, it’s sort of a barrier to doing that referral, because I know if I make the referral to palliative care service aggressively and it is other than symptoms, you don’t need us.So,it’s atwo-way problem” (P4.5)
Palliative care role not always clear to hepatologists	“If I’m completely honest I don’t actually know exactly what they do. I know that they manage symptoms and expectations and family and stresses and things like this” (P1.1)
Referral process convoluted	“I guess one of the other challenging things is when you complete the referral online, it takes up to half an hour to complete. And then not only that, you have to ring them up and make sure they got it, email so it’s almost like a three step process to make sure it’s actually been accepted and then you have to wait so once you put in your referral thing you have to wait a while to find out if they’ve been accepted by that space and if it’s documented properly” (P1.2)
Need for clinician training in HCC management	“I think there are some skills that I think most gastroenterologists can sense what’s a bad livercancer,but I think perhaps they do need to be more formal teaching around specific system management and specific medications you use and all those sorts of aspects because it’s also probably a little bit naive to think that you know everything” (P2.2)
Need for clinician training in palliative care	“There are still a lot of treating clinicians that only feel like palliative care referral is warranted when patients are actively dying. Educating the community and clinicians about involving palliative care services early in patients with incurable illness that has a high symptom burden” (P3.5)

All participants commented on the referral pathway within their respective networks and highlighted the lack of clarity regarding patient eligibility criteria and timing of referrals, whilst calling for clear guidelines to support the referral process. Many individuals and particularly the hepatology clinical nurse consultants discussed the convoluted administrative processes involved in referring patients to palliative and supportive care. Referrals were not always straightforward to make and navigating the different and varied individual systems and models under which palliative care services operated within local health districts added to the complexity of making a referral.

All respondents acknowledged a lack of education and training in providing palliative and supportive care to liver cancer patients, and some felt that a greater understanding of the roles of palliative care clinicians and the services they provide would help them decide when to refer. More specifically, they expressed a lack of knowledge regarding specific palliative care pharmacotherapies that could be utilised in patients and a lack of training in patient and family discussions on ‘breaking bad news’, ‘prognosis’, and ‘palliative care discussions’. This limited knowledge stemmed from limited educational opportunities about palliative and supportive care among gastroenterologists/hepatologists and limited collaboration between the two specialties. Historical viewpoints on palliative care which only encompassed the end-of-life care when patients prognosis is measured in days were expressed when discussing their colleagues’ perceptions of the work of palliative and supportive care, though no participants in this study expressed this viewpoint. Conversely, participants also felt that palliative care clinicians may not be fully aware of the natural progression and clinical management of HCC, and they too needed education to provide optimal care for patients with HCC. This is illustrated by respondents describing that their referred HCC patients were not accepted into palliative care services because of a perception that the patients were receiving ongoing active management and a lack of understanding that the patient’s trajectory to ultimate demise would not change.

All respondents reported that the current guidelines for patients with HCC provided little clarity on which patients should be referred, and a fair number of practitioners were operating outside the guidelines when considering early referral to palliative care. Some participants discussed their opinions regarding the perceived benefit of early palliative care referral in HCC patients, despite the lack of evidence to guide practice. Participants noted that symptoms discovered by themselves or by nursing staff alerted them to the potential need for palliative and supportive care input, using symptom burden as a rationale for early referral to palliative and supportive care. However, several mentioned that waiting until a significant symptom burden is apparent may not result in the best outcomes for patients, and many felt that earlier referral was needed.

Many healthcare professionals reflected that they had no clear understanding of the specific role that palliative care played in the management of patients with HCC, nor were a number of participants aware of the specific outcomes of referral or how to alter it (i.e. whether the patient is seen by a palliative care physician, clinical nurse consultant, or usual community palliative care services).

### Theme 4: clinician perceptions about patients’ attitudes to palliative care

All participants detailed interactions with patients where there was limited understanding of what benefits palliative care can provide for HCC patients. This was thought to be due to a general perception in the community that palliative care is for end-of-life treatment only.

Many respondents had experienced difficulties regarding CALD populations and their understanding of palliative care and cultural norms, where certain cultural groups simply do not discuss death and dying. Individuals acknowledged that their education in this area is lacking and that it is difficult to assess a patients’ understanding of their clinical situation, even when interpreters are involved. Some participants acknowledged the usefulness of facilitators described in Table 5, for HCC patients in accessing and discussing palliative and supportive care. Education materials about HCC were available at each of the districts, but the healthcare workers stated there was little material on palliative and supportive care in these educational materials. Whilst some of this material was covered during in-person consultations, discussing palliative care was often avoided because of the negative perceptions that HCC patients and their families had of the topic.

**Table 5:** Theme 4: clinician perceptions about patients’ attitudes to palliative care

Subthemes	Illustrative quote(s)
Patients limited understanding of palliative care	“Quite often palliative care is seen as sort of terminal management for patients and therefore, there seems to be a bit of reluctance from the specialists as well as from the patients to actually see palliative care nurses or, doctors” (P2.4)
Cultural barriers to accepting palliative care *Language appropriate info*	“Culturally it can be a barrier because some cultures really don't want to talk about end of life and when you talk about palliative care that opens a Pandora box” (P2.1) “Well, a lot of them obviously don’t speak English so even though you can get interpreters it’s just not you know it’s not as convenient as speaking to the patients directly and telling them what’s available etc. And often, we get the family members to interpret for the patient. So, whether something is lost in translation, it’s difficult to know” (P1.7)
Facilitators of patient in palliative care	“The use of a helpline I’ve already mentioned they have our Clinical Nurse Consultant’s direct number” (P4.4)
Information, education for patients needed *Changing perceptions of palliative care* *Information is given to patients* *Information not always given to patients* *Need patient trust of palliative care benefits*	“There is like a very small area of our booklet, as well as our website, which is the Liver Wellness Program website, that has some mention of it. But I have my doubts that the patients access it” (P1.6) “Just rebranding it to completely supportive care and not calling it palliative care at all. I think the district can try to raise the profile of it” (P2.2) “We have an extensive in-house education and support service. Focuses on both the physical and psychosocial needs of the patients which goes beyond the actual liver cancer treatment” (P2.6) “We don’t have any pamphlets. In the beginning of it anyway and if we had any, which we don’t, we don’t give anything to the patients any sort of material” (P4.5) “…you need to build that rapport and that trust before you then broach the subject” (P1.4)
Limited patients’ capacity for self-care and care options *Patients need support with appointments*	“Some people, for instance, if they aren’t surveillance for chronic liver disease, they get sick of the process every 6 months. ‘Come on I’ve done it for 5 years. I’m sick of it. Nothing happened. Why do I have to do this? And you know, it’s just okay’” (P3.4) “I probably need access to find them to confirm all their appointments because some of them seem to just have problems with their appointments in general and follow up…” (P1.2)
Patient acknowledgement of fatal nature of disease	“Sometimes they don’t even have the understanding that cirrhosis means their liver has not been functioning very well. They think if they’re coming to the liver clinic and they’re seeing a doctor every six months and they’re doing what they have been asked to do like the blood tests it means they are okay” (P1.6)
Patient and family anxiety, stress, fear *Carer and family have better insight*	“I think the main thing is, does this mean I’m dying. I think that’s the main question” (P4.3) “What does this mean? When am I going to die? Are you going to stop treating me? How long have I got? Is the other good one” (P4.6) “And having family members present is also very important. So, everyone not just the patient, but everyone, in the family or their support people all on the same page. So, there is no confusion” (P2.4)
Patient not ready for palliative care	“And then, even when we try to touch on the subject, it’s a tough one and you know they’re not always accepting” (P3.2)
Patient ready for palliative care	“In some cases, I have to say there has been a lot of relief from the patient and sometimes the family but more so from the patients when you do bring out palliative care as a, as a concept and as a service” (P1.4)

Participants expressed concern over the patients’ and families’ poor health literacy which limited a patient’s capacity for self-care and care options. Expanding on this poor health literacy about HCC, many participants found that patients were unaware of the fatal nature of their underlying disease, despite previous conversations. This compounded the patients’ and families’ stress, anxiety, and fear when discussing referral to palliative and supportive care as the topic sometimes came as a surprise. All healthcare workers had had negative interactions with patients and their families who expressed concerns and limited understanding when discussing palliative and supportive care. Mitigating this understanding from both a patient and family perspective was imperative for participants to be able to successfully refer patients to palliative care and to have these services accept HCC patients into the service.

Many respondents had dichotomous views on previous patients’ interactions regarding readiness for referral to palliative care. Participants reported that some patients had difficulty discussing referral to palliative care and were not prepared to accept the referral. However, some patients that the health professionals pre-emptively perceived as being reluctant to accept referral to palliative care were quite accepting and viewed the referral with relief.

## Discussion

Palliative and supportive care integration into cancer care pathways is becoming the standard of care throughout the world [8, 11, 12]. This study addressed a knowledge gap about health professionals’ experiences and perceptions of the potential value of integrating palliative and supportive care in HCC management. The major findings from this study detailed an often confusing and heterogeneous environment with major barriers to implementing and providing palliative and supportive care to HCC patients from a societal, organisational, cultural, and resource perspective. Gaps in educational opportunities and limited best practice guidelines to inform clinical decisions about referring patients to palliative care were identified. The study also identified a perceived limited understanding among palliative care clinicians of the nuanced care needs of patients with HCC.

One of the main barriers that participants perceived exists within the system regarding HCC and palliative and supportive care is the lack of referral pathways, with a lack of underpinning evidence-based guidelines. Current international guidelines in hepatology are at odds with guidelines from oncology [11, 25–27]. A recent review detailed the limited evidence base and the future implications for practice [28]. Without an appropriate evidence base, the implementation of palliative and supportive care in HCC is difficult due to the non-linear natural history of the disease coupled with the heterogeneous application of treatment modalities based on institutional expertise. The ability to perform research into implementation of palliative and supportive care as a complex healthcare intervention with multidisciplinary adaptation in behaviours and process normalisation within resource constrained environments is thus difficult [29]. This coupled with a lack of discrete funding pathways hampers the ability to implement empirical evidence into the care-space, which consequently impairs the ability of health professionals to advocate for earlier referral to palliative care.

One of the most consistent themes identified was the perceived benefit of palliative care medicine for patients with liver cancer [22]. All participants detailed positive experiences that resulted from the input of palliative and supportive care medicine for referred patients. There is an inherent level of trust in palliative and supportive care medicine to provide care and services to the HCC population, despite a lack of knowledge among hepatologists about what exactly is provided by these services. The expansion of palliative and supportive care services is a relatively new discipline, which evolved from pain management at end of life [30]. The broadening of the palliative care services skill set beyond this scope has been well documented within the speciality but has failed to be communicated beyond existing silos [30]. The inherent trust that has been borne out of this area to the specialists involved is likely from patients’ perceived benefit from the care input, which is communicated back to the referring hepatologists. Whilst this phenomenon of patient attitudes towards services displays sociological aspects of collectivistic culture in terms of a relationship oriented, interdependent, holistic thinking style focusing on group harmony, the application to the complexity of interactions in referral behaviours between medical specialities has not been highlighted [31, 32].

Patient and family level barriers to palliative and supportive care in liver cancer as perceived by clinicians were explored in this study. These findings were consistent with the literature in other cancer types when discussing palliative and supportive care [33–38]. One factor highlighted by participants was the level of patient and family anxiety and fear, limiting acceptance of referral. All healthcare workers expressed concerns that the offer of palliative care would raise preconceived perceptions of attitudes among patients and carers, including perceptions that death is imminent, and that active treatment will be withdrawn [30]. This was compounded by a perception of poor health literacy by the respondents; however, this has not been explored with patients and carers, who’s viewpoints would add clarity to this picture. Healthcare literacy in palliative care consists of a complex interplay between patient, carer, clinician, and system issues. Targeted interventions providing end-of-life care support has been shown to improve end-of-life care literacy and could addressed the gaps in communication between clinician, patient, and carers [39].

Another barrier identified by clinicians included the specific difficulties experienced when engaging and discussing palliative care with CALD populations. As large numbers of liver cancer patients in Australia come from CALD backgrounds, these populations need culturally appropriate communication methods and patient education resources to address these barriers [40]. Communication surrounding palliative care and its benefits has been shown to be more difficult in culturally and linguistically diverse communities compared to their Anglo-Saxon counterparts [41]. Rebranding ‘palliative care’ to ‘supportive care’ to enable early integration into cancer treatment pathways has been considered and endorsed by governing societies; however, the roll-out into the broader healthcare environment has not been fully realised [30, 42]. Suggested insights from participants at the one site who had integrated palliative care had removed this connotation by rebranding it ‘supportive care’ and had significantly decreased patient associated barriers to acceptance of referral.

One of the most common barriers to provision of palliative care elucidated in this study was the lack of education and training regarding various aspects of supportive care for healthcare professionals who manage patients with HCC. This was not limited to one area within this domain but encompassed symptom management and communication with patients surrounding the underlying fatal nature of their disease. This generalisation is not limited to healthcare professionals providing HCC care and is prevalent across several specialities as demonstrated in the literature [33, 35, 38, 43].

## Strengths and limitations

This study was conducted across four major public tertiary centres providing interdisciplinary liver cancer care in NSW, Australia. Thus, the results may not be representative of all health professionals who provide care to patients with HCC. It is important to note that this may not precisely represent the perspectives of healthcare professionals predominantly situated in private sector medicine. The four tertiary centres are in metropolitan Sydney, but all four accept referrals from across the state of NSW. Nevertheless, the experiences of gastroenterologists, hepatologists, and palliative care staff who work in regional centres were not captured, and this should be a priority for the future. The findings are context-specific to the four clinics which operate under the constraints of the Australian health system. Therefore, the results may not be transferable to other settings internationally.

We employed methodological approaches to mitigate the likelihood of bias in the interpretation of the qualitative data. This included analysis by skilled qualitative researchers who were independent of the data collection process and were not involved or affiliated with the four liver clinics, double-coding and inter-coder agreement testing and conflict resolution, and critical reflection and expert discussion.

## Future research

The remit of palliative and supportive care for patients with HCC has many unique attributes as the disease occurs most frequently in the setting of cirrhosis. Hence, a large proportion of patients are managed during their entire disease course by liver specialists who have unique expertise in the management of cirrhosis and its complications. To realign with the current manifest needs, there needs to be significant research investment to (a) further establish perspectives and attitudes towards palliative care in liver cancer with an endeavour to capture patients and carers viewpoints, (b) establish earlier setpoints for initiation of palliative care referral not just for patients with liver cancer but also for those with end-stage liver disease, (c) define what these set points are for this specific population, and (d) increase education of healthcare providers on the roles and benefits of palliative and supportive care in the liver cancer patient journey.

## Conclusion

There is limited integration of hepatology services with palliative care services and limited referral pathways, compounded by the complexity created by different palliative care services and their acceptance criteria and geographical boundaries. Clinical guidelines that provide evidence-based recommendations regarding the timing of referral and the specific palliative care needs of patients with HCC are needed to optimise care. There is a need for interdisciplinary education opportunities to upskill liver cancer teams in palliative care while at the same time upskilling palliative care teams in the management of HCC. Clinicians experienced significant stigma and limited understanding of the role of palliative care for their patients, especially for those from CALD populations. Targeted accessible patient information resources in multiple community languages and in multiple formats should be co-designed and distributed through liver clinics and in primary care settings.

## Data Availability

No datasets were generated or analysed during the current study.
